# Regulation of Apoptotic Cell Clearance During Resolution of Inflammation

**DOI:** 10.3389/fphar.2019.00891

**Published:** 2019-08-13

**Authors:** Simone Arienti, Nicole D. Barth, David A. Dorward, Adriano G. Rossi, Ian Dransfield

**Affiliations:** Centre for Inflammation Research, Queen’s Medical Research Institute, University of Edinburgh, Edinburgh, United Kingdom

**Keywords:** apoptosis, phagocytosis, inflammation, resolution, macrophage

## Abstract

Programmed cell death (apoptosis) has an important role in the maintenance of tissue homeostasis as well as the progression and ultimate resolution of inflammation. During apoptosis, the cell undergoes morphological and biochemical changes [e.g., phosphatidylserine (PtdSer) exposure, caspase activation, changes in mitochondrial membrane potential and DNA cleavage] that act to shut down cellular function and mark the cell for phagocytic clearance. Tissue phagocytes bind and internalize apoptotic cells, bodies, and vesicles, providing a mechanism for the safe disposal of apoptotic material. Phagocytic removal of apoptotic cells before they undergo secondary necrosis reduces the potential for bystander damage to adjacent tissue and importantly initiates signaling pathways within the phagocytic cell that act to dampen inflammation. In a pathological context, excessive apoptosis or failure to clear apoptotic material results in secondary necrosis with the release of pro-inflammatory intracellular contents. In this review, we consider some of the mechanisms by which phagocytosis of apoptotic cells can be controlled. We suggest that matching apoptotic cell load with the capacity for apoptotic cell clearance within tissues may be important for therapeutic strategies that target the apoptotic process for treatment of inflammatory disease.

## Apoptotic Cell Clearance in Homeostasis and Inflammation

The controlled elimination of cells during development allows the remodeling of tissues and organs to purpose them for specialized functions (reviewed in Elliott and Ravichandran, 2010). In fully formed adult organisms, it is estimated that approximately 50 million cells are deleted by apoptosis every hour, providing a mechanism for homeostatic self-renewal (Nagata, 2018). Apoptosis also has an important role in the responses to injury or infection, controlling the numbers of inflammatory cells present at inflamed sites (Poon et al., 2014), shaping the repertoire of effector lymphocyte populations (Albert, 2004), and determining the capacity for repair and regeneration of tissue following injury (Bosurgi et al., 2017). Importantly, the consequences of cell death are ultimately defined by the mechanism(s) by which apoptotic cells (AC) are removed from tissues (Savill et al., 2002). Thus, the molecular pathways involved in recognition and subsequent phagocytosis of AC will determine whether apoptotic cell clearance is immunologically “silent” or even anti-inflammatory. By implication, breakdown or failure of normal AC removal mechanisms will increase the tissue load of AC and secondarily necrotic cells and has the potential to drive the aberrant tissue repair responses and failure to restore tissue integrity (DeBerge et al., 2017b).

The efficient clearance of AC from tissues requires that AC are specifically recognized and destroyed, either by neighboring cells or by specialized phagocytes (Fond and Ravichandran, 2016). Although AC retain plasma membrane integrity, alterations in composition of the membrane lipids, carbohydrates, and proteins provide molecular cues that mark them for recognition by other cells. In particular, translocation of anionic phospholipids [phosphatidylserine (PtdSer) and phosphatidylethanolamine] from the inner leaflet to the outer leaflet of the plasma membrane represents one of the hallmarks of apoptosis (Segawa and Nagata, 2015). Exposure of PtdSer on the outer leaflet of the plasma membrane can be detected directly *via* specific PtdSer receptors (see below). Alternatively, binding of proteins that act to “opsonize” the AC membrane enables indirect engagement of additional receptor pathways for the recognition and internalization of AC (Stitt et al., 1995; Païdassi et al., 2008). Additional changes in glycosylation (Hart et al., 2000; Franz et al., 2006), crosslinking (Piacentini et al., 1991), or proteolytic shedding of proteins (Dransfield et al., 1994) on the AC surface provide additional “apoptotic cell associated molecular patterns” that also influence AC recognition (Franc et al., 1999). The repertoire of phagocyte receptors that are engaged during recognition and subsequent internalization of AC may determine the subsequent response of the phagocytic cell.

## Molecular Mechanism of AC Clearance

There are many different receptor families involved in the process of phagocytosis of AC [extensively reviewed elsewhere (Elliott and Ravichandran, 2010; Freeman and Grinstein, 2014; Nagata, 2018; Lemke, 2019), summarized in **Table 1**]. Genetic deletion of a single receptor pathway seldom eliminates phagocytic clearance capacity, suggesting a level of functional redundancy in AC clearance, both *in vitro* and *in vivo*. AC clearance pathways may have multiple, partially overlapping physiological roles, as the extent to which specific deletion impacts upon homeostasis and immune processes *in vivo* differs (Gregory and Devitt, 2004). However, adaptation to universal gene deletion may complicate interpretation and studies of inducible knockouts will provide important additional insights into the role of specific molecular pathways that are involved in AC clearance *in vivo*.

Receptors mediating AC phagocytosis can be broadly divided into non-opsonic (direct recognition) or opsonic receptors (indirect recognition). The receptors mediating AC clearance can be further categorized based on their potential for transducing signals that control the internalization of AC (Barth et al., 2017). For example, although T cell immunoglobulin and mucin domain containing 4 (TIM4) and brain-specific angiogenesis inhibitor 1 (BAI-1) are both capable of mediating recognition of PtdSer, only BAI-1 is capable of directly mediating signal transduction (Park et al., 2007; Park et al., 2009). For a single phagocyte, efficient phagocytosis may require cooperative activity of receptors involved in AC clearance. Optimal phagocytic responses may require the establishment of a phagocytic synapse with spatial co-localization of molecules of similar dimensions at the interface between phagocyte and target, together with exclusion of phosphatases such as CD45 or CD148 (Barth et al., 2017).

**Table 1:** Summary of key molecular pathways mediating apoptotic cell phagocytosis. Examples of molecules mediating either direct or indirect (*via* bridging molecules) recognition of apoptotic cells, together with putative signaling mechanisms that are triggered (Savill et al., 1990; Savill et al., 1992; Stitt et al., 1995; Mevorach et al., 1998; Taylor et al., 2000; Albert et al., 2000; Scott et al., 2001; Stuart et al., 2007; Park et al., 2007; Rothlin et al., 2007; Park et al., 2008; Tibrewal et al., 2008; Païdassi et al., 2008; Park et al., 2009; Nakahashi-Oda et al., 2012; Ramirez-Ortiz et al., 2013; Kourtzelis et al., 2019).

Receptor	Ligand	Signaling
**Direct recognition**		
BAI-1	PtdSer	GPCR – DOCK180, ELMO, Rac-1 (Park et al., 2007)
TIM-4	PtdSer	Indirect *via* integrins (Park et al., 2009)
CD300	PtdSer	ITIM (Nakahashi-Oda et al., 2012)
Stabilin-2/MEGF-10	PtdSer	*via* GULP (Park et al., 2008)
**Indirect recognition**		
MER	PROS1, GAS6 (Stitt et al., 1995)	Autophosphorylation, Akt, PLCγ2, FAK, Rac-1 (Tibrewal et al., 2008)
AXL	Gas6	IFNAR, STAT1, SOCS1/3 (Rothlin et al., 2007)
SCARF (Ramirez-Ortiz et al., 2013)	C1q (Païdassi et al., 2008)	
Integrin αMβ2 (Mevorach et al., 1998)	C1q	
Integrin αvβ5	MFG-E8 Del-1	FAK, DOCK180, Rac-1 (Akakura et al., 2004; Albert et al., 2000)
Integrin αvβ3	MFG-E8 Del-1 TSP-1	CRKII, DOCK180, Rac-1 (Hanayama et al., 2002; Savill et al., 1990; Savill et al., 1992)
CD36	TSP-1	Fyn, Pyk2 (Stuart et al., 2007)

(Savill et al., 1990; Savill et al., 1992; Stitt et al., 1995; Mevorach et al., 1998; Albert et al., 2000; Hanayama et al., 2002; Akakura et al., 2004; Park et al., 2007; Rothlin et al., 2007; Stuart et al., 2007; Païdassi et al., 2008; Park et al., 2008; Tibrewal et al., 2008; Park et al., 2009; Nakahashi-Oda et al., 2012; Ramirez-Ortiz et al., 2013)

## Regulation of Apoptotic Cell Clearance

Phagocytosis of AC may be regulated rapidly (within minutes) in response to exogenous or microenvironmental signals *via* changes in the ligand binding activity of receptors. Alternatively, the spatial distribution of receptors that mediate AC uptake may result in the formation of receptor micro-clusters that facilitate phagocytosis, as has been demonstrated for FcgR (Lopes et al., 2017). Phagocytosis of AC may also be controlled over a more prolonged time frame *via* changes in the repertoire of receptors that are expressed on the phagocyte membrane. For receptors that recognize AC *indirectly* through “bridging” ligands that bind to the AC, the availability of those ligands represents another level of control. Increased AC phagocytosis has been shown to occur following crosslinking of CD44 (Hart et al., 2012) or in the presence of soluble mediators such as galectin-3 (Caberoy et al., 2012); here we consider some of the key factors that exert control of AC phagocytosis and whether the underlying mechanisms of regulation could be exploited for therapeutic gain (summarized in **Table 2**).

**Table 2:** Summary of key mechanisms by which apoptotic cell phagocytosis is regulated. Examples of mediators that act to regulate phagocytosis of apoptotic cells, including putative mechanisms that are involved in regulation.

Regulatory pathway	Mechanism
LipoxinA4	FPR mediated activation of myosin IIa, Rho, Rac-1, cdc42 (Reville et al., 2006; Maderna et al., 2010)
Resolvin E1 (RvE1)	ERV-1/ChemR23 (Akt and ribosomal S6 protein phosphorylation) (Pirault and Bäck, 2018; Ohira et al., 2010), *BLT1 sequestration (Arita et al., 2007)
D series Resolvins (D1, D2, D3, D5)	GPR32, GPR18 (PKA, STAT3), ALX/FPR2 (Pirault and Bäck, 2018; Krishnamoorthy et al., 2010)
Maresin 1 (MaR1)	*Inhibition of LTAH_4_ (Dalli et al., 2013)
Del-1	RGD-dependent binding to integrins (Kourtzelis et al., 2019)
Osteopontin	Competition for integrin ligand binding (Sakamoto et al., 2016)
Fibronectin	Scaffold for TIM-4 (Lee et al., 2018), β1 integrin-dependent signaling (McCutcheon et al., 1998)
Fibronectin and CD31	Activation of integrin α5β1 and FN-dependent uptake (Vernon-Wilson et al., 2006)
Glucocorticoids	Upregulation of MER expression and activation (McColl et al., 2009; Zagórska et al., 2014)
Glucocorticoids	Cytoskeletal regulation (Rac-1) (Giles et al., 2001)
LXR agonists	Upregulation of phagocytic receptors inc. Mer (A-Gonzalez et al., 2009)
	
CD44 cross-link	Cytoskeletal regulation (Hart et al., 2012), Membrane picket formation (Freeman et al., 2018)
CD14 cross-link	Mer phosphorylation (Zizzo and Cohen, 2018)
Galectin-3	Cytoskeletal regulation (Erriah et al., 2019)
CD47	SIRPα–ITIM mediated SHP1 and SHP2 activation (Barclay and Van den Berg, 2014; Okazawa et al., 2005)

*Additional anti-inflammatory effects (Hart et al., 1997; McCutcheon et al., 1998; Godson et al., 2000; Giles et al., 2001; Okazawa et al., 2005; Reville et al., 2006; Vernon-Wilson et al., 2006; A-Gonzalez et al., 2009; McColl et al., 2009; Ohira et al., 2010; Hart et al., 2012; Krishnamoorthy et al., 2012; Zizzo et al., 2012; Barclay and Van den Berg, 2014; Zagórska et al., 2014; Sakamoto et al., 2016; Freeman et al., 2018; Laguna-Fernandez et al., 2018; Lee et al., 2018; Pirault and Bäck, 2018; Zizzo and Cohen, 2018; Erriah et al., 2019; Kourtzelis et al., 2019).

## Lipid-Derived Mediators

**Figure 1:**
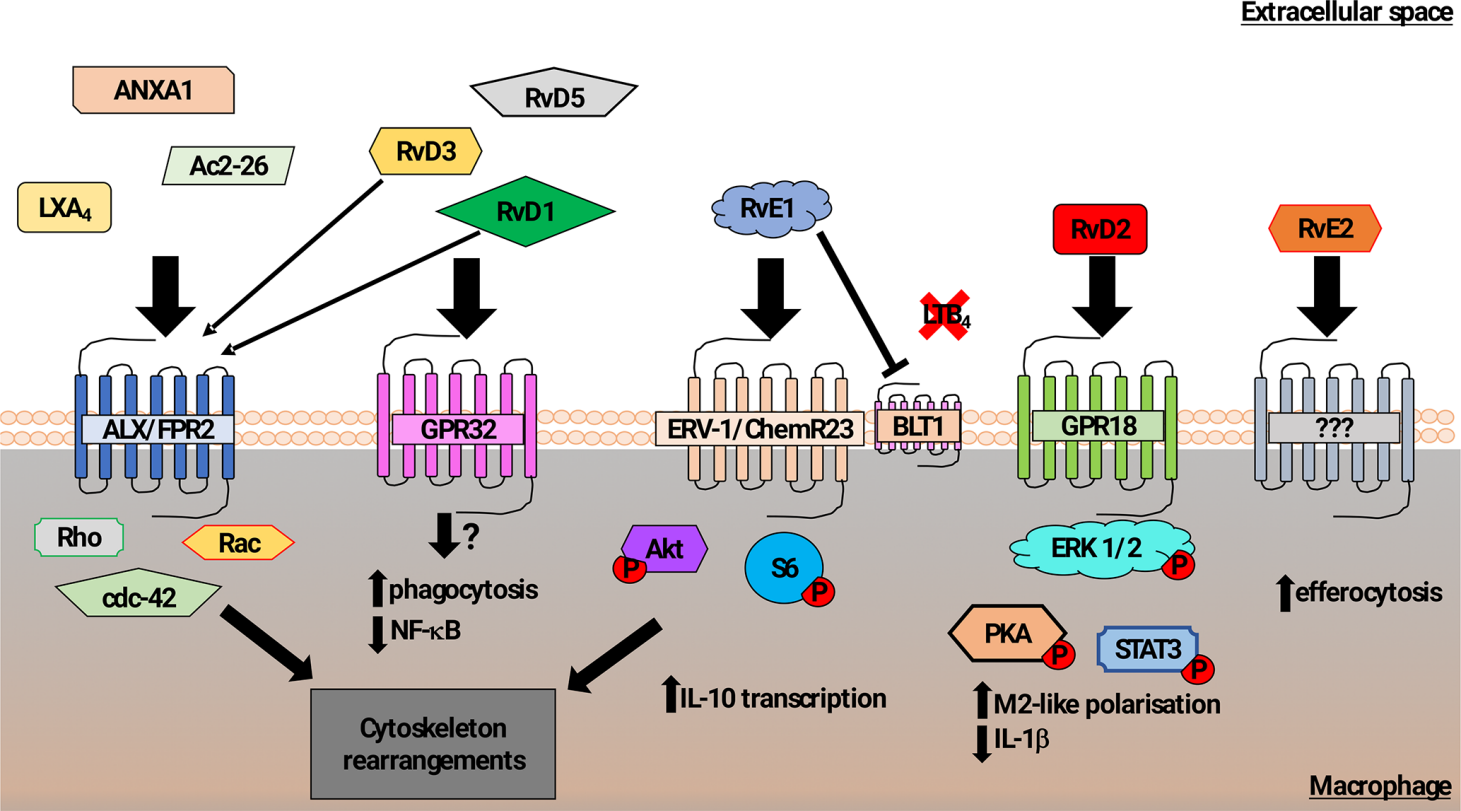
Schematic representation of mechanisms promoting macrophage phagocytosis of AC by lipid mediators. In addition to a role in recognition of N-formylated peptides that are generated during bacterial and mitochondrial protein synthesis, the formyl peptide receptor 2 (ALX/FPR2) also binds to Lipoxin A4, Annexin A1, and Ac2-26 (N-terminal part of Annexin A1) to increase macrophage phagocytosis of AC (Godson et al., 2000; Maderna et al., 2002; Scannell et al., 2007; Maderna et al., 2010). Signaling following ALX/FPR2 binding by these ligands was shown to induce rearrangements in the actin cytoskeleton in a Rho, Rac, and cdc42-dependent manner. GPR32 is thought to be the main receptor for the resolvin D family members 1, 3, and 5 (RvD1,3,5) that acts to promote phagocytosis of AC and reduce NF-κB activity. RvD1 and RvD3 were also found to bind ALX/FPR2 with high affinity and induce macrophage phagocytosis (Arita et al., 2007; Krishnamoorthy et al., 2010, Krishnamoorthy et al., 2012; Dalli et al., 2013b). The resolving E family member 1 (RvE1) increased macrophage phagocytosis of AC *via* binding to ERV-1/ChemR23. Phosphorylation of Akt and S6 proteins induced cytoskeletal rearrangements as well as promotion of transcription of the anti-inflammatory cytokine IL-10 (Laguna-Fernandez et al., 2018). RvE1 can also competitively bind to the leukotriene B4 receptor BLT4, acting to reduce pro-inflammatory signaling (Arita et al., 2007; Ohira et al., 2010; Laguna-Fernandez et al., 2018). Resolvin D2 (RvD2) mediated activation of GPR18 induced an M2-like macrophage phenotype exhibiting increased phagocytosis *via* a mechanism involving phosphorylation of the ERK, PKA, and STAT3 (Fredman and Serhan, 2011; Dalli et al., 2013b). Although resolving E2 (RvE2) was also reported to induce macrophage phagocytosis, the pathway that controls this effect has not been clearly identified (Oh et al., 2012).

Multiple studies have demonstrated the contribution of lipid mediators to the control of phagocytosis, shown schematically in **Figure 1**. The lipoxin family of lipids, derived from arachidonic acid, were shown to have anti-inflammatory effects that impact on the resolution phase of inflammation. Lipoxin A4 (LXA_4_) stimulates phagocytosis of AC (Godson et al., 2000), acting *via* the G-protein coupled receptor (GPCR) formyl peptide receptor 2 (FPR2) to induce Rho, Rac, cdc42-dependent actin-cytoskeleton rearrangements (Maderna et al., 2010). Both FPR1 and FPR2 confer recognition of *N*-formylated peptides generated during bacterial and mitochondrial protein synthesis and are abundantly expressed on leukocytes. Although FPR1 signaling activates pro-inflammatory signaling (Dorward et al., 2015), certain FPR2 agonists elicit anti-inflammatory responses in innate immune cells (e.g., LXA_4_, Annexin A1, Ac2-26) (Scannell et al., 2007; Filep, 2013), suggesting a specific role for FPR2 in controlling phagocyte responses during resolution of inflammation. Interestingly, in a mouse model of arthritis, genetic deletion of FPR2 abrogated the pro-resolving effects of RvD3 (Arnardottir et al., 2016).

The resolvin family of anti-inflammatory/pro-resolution lipids derived mainly from omega-3 fatty acids, especially eicosapentaenoic acid (EPA) and docosahexaenoic acid (DHA), includes the E-series of the anti-inflammatory lipid mediators, resolvin-E1 (RvE1) and resolvin-E2 (RvE2) (Kohli and Levy, 2009), and the D-series resolvins (Gilroy et al., 2004). These mediators actively promote resolution of inflammation *via* a number of mechanisms, including down-regulation of NF-κB signaling and dampening the effects of pro-inflammatory mediators (Arita et al., 2007). Resolvins also promote phagocytosis by binding to various GPCRs. RvE1 binding to ChemR23 on macrophages enhances macrophage phagocytosis of zymosan particles *via* a mechanism involving enhanced Akt and ribosomal protein S6 phosphorylation (Ohira et al., 2010; Laguna-Fernandez et al., 2018).These effects are similar to the rapid increase in phagocytosis of AC and zymosan particles that is induced by the natural peptide ligands for ChemR23 such as chemerin (Cash et al., 2010). Similarly, RvE2 has also been reported to promote phagocytosis of zymosan, though it may act through a different repertoire of GPCR from RvE1 (Oh et al., 2012).

The resolvin D-series (RvD1-5) lipids have also been demonstrated to have anti-inflammatory effects on leukocyte recruitment and production of anti-inflammatory cytokines, including IL-10. RvD lipids also act to increase phagocytosis *via* the action of different GPCR (Pirault and Bäck, 2018). RvD3 and RvD1 act *via* GPR32 to increase macrophage phagocytosis of AC and zymosan. RvD1 also promotes phagocytosis *via* the FPR2 (Krishnamoorthy et al., 2012), which had been shown to regulate AC clearance by lipoxin A4 (Maderna et al., 2010). Macrophage phagocytosis of zymosan, *E. coli* (Spite et al., 2009), and AC was increased by RvD2 both *in vitro* and *in vivo* (Chiang et al., 2015). Analysis of downstream signaling suggested a mechanism in which RvD2 acted on GPR18 to regulate PKA and STAT3 (Fredman and Serhan, 2011; Dalli et al., 2013a; Chiang et al., 2017).

Another class of specialized pro-resolving lipid-derived mediators that also modulate macrophage phagocytosis of AC are the maresins (Serhan et al., 2009). In addition to increasing macrophage phagocytosis of AC in a similar manner to RvD1, maresin 1 (MaR1) was also shown to reduce neutrophil infiltration and to promote tissue regeneration as well as inhibiting leukotriene A_4_ hydrolase (LTA_4_H) activity, shifting macrophages toward a pro-resolution phenotype (Dalli et al., 2013c).

In summary, the activity of specialized pro-resolving lipid-derived mediators (SPM) activity peaks during the resolution phase of inflammation. High affinity interactions between different SPM and their receptors (e.g., RVD1/3-ALX/FPR2) suggest that there may be synergistic activity of SPM during the resolution phase of inflammation. In terms of regulation of phagocytic function, specific SPM-receptor interactions lead to the phosphorylation of proteins that are involved in the regulation of cytoskeletal organization that are also required for cell migration. As a consequence, neutrophil transmigration was reduced following treatment with SPM (e.g., RvD1, MaR1) together with increased evidence of tissue regeneration. In addition, inhibition of the pro-inflammatory effects of LTB_4_ mediated by the LTB_4_ receptor, BLT1, was induced by RvE1 and MaR1. Thus, the effects of pro-resolving lipid mediators are not restricted to phagocytosis of AC, in keeping with a broader role during resolution of inflammation and restoration of tissue homeostasis.

## Extracellular Matrix—Integrins and Their Ligands

A role for integrin αVβ3 in AC clearance by macrophages was first demonstrated in 1990 (Savill et al., 1990), and induction of expression of αV (Andreesen et al., 1990) and partnering β subunits [β3 (Savill et al., 1990) and β8 (Kumawat et al., 2018)] during *in vitro* macrophage differentiation confers the capacity for uptake of AC *via* bridging factors such as thrombospondin-1 or MFG-E8 (Akakura et al., 2004). Another αV ligand that promotes phagocytosis of AC and acquisition of a pro-resolving macrophage phenotype is Del-1, an RGD-containing secreted molecule (Kourtzelis et al., 2019) that binds to AC (Hanayama et al., 2002). Deletion of Del-1 results in reduced expression of many genes associated with regulation of inflammation including liver X receptor (LXR), TGF-β1, ATP-binding cassette transporter 1 (ABCA1), transglutaminase 2, Axl, CD36, and uncoupling protein 2. Integrin-dependent phagocytosis of AC can be inhibited by addition of soluble αVβ3 ligands (fibronectin or vitronectin) (Savill et al., 1990), or direct competitors of αV ligand binding such as HMBG1 (Friggeri et al., 2010). Molecules that bind directly to αVβ5 such as histone H3 also inhibited AC phagocytosis (Friggeri et al., 2012).

However, rapid regulation of integrin-dependent cellular interactions may also occur as a result of outside-in signaling (Hogg et al., 1993). Integrin-dependent signaling also regulates AC phagocytosis. For example, osteopontin acts to block αV/MFG-E8-mediated engulfment *via* prevention of dissolution of the actin cup that is formed beneath bound apoptotic targets, thereby prolonging diffuse Rac activation (Sakamoto et al., 2016). Inhibition of integrin-dependent Rac1 or ROCK signaling was associated with reduced phagocytosis of ACs and fibronectin-coated beads in mice lacking α8 integrins, with delayed healing in a model of glomerulonephritis (Marek et al., 2018). Association of TIM4 with αVβ3 acts to potentiate AC phagocytosis. Fibronectin was identified as a TIM-4 binding partner, providing a scaffold to bring TIM-4 and integrins together to facilitate phagocytosis (Lee et al., 2018). Consequently, disruption of TIM-4 interaction with fibronectin causes a reduction in TIM-4-dependent phagocytosis, possibly as a result of altered integrin signaling (Flannagan et al., 2014). Albert and colleagues demonstrated that aVβ5 formed a complex with Crk/DOCK180 and Rac, homologues of the key phagocytic module (Ced-2, 5 and 10) identified in *C. elegans* (Albert et al., 2000).

Other integrins may also be important regulators of AC phagocytosis. Increased phagocytosis of ACs was observed following adhesion to extracellular matrix ligands in a manner that was partially dependent on b1 integrin activity (McCutcheon et al., 1998). The extent of integrin-dependent adhesion and signaling may be critical, since interaction with extracellular matrix modified by cigarette smoke resulted in reduced AC clearance, possibly due to sequestration of integrins involved in phagocytosis or cytoskeletal regulation (Kirkham et al., 2004; Minematsu et al., 2011; Tran et al., 2016). Similarly, reduced phagocytosis and pro-inflammatory cytokine production were reported following exposure of macrophages to bushfire smoke extract (Hamon et al., 2018). Fibronectin may also have an important role in the selective engulfment of AC following CD31 ligation. Although CD31 mediates tethering of both AC and viable cells, CD31-dependent activation of phagocyte α5β1 facilitated specific phagocytosis of ACs *via* a fibronectin bridge (Vernon-Wilson et al., 2006).

Integrins also have a key role in the regulation of phagocytosis by controlling the localization of key molecules such as the phosphatase, CD45 (Freeman et al., 2016). In their elegant studies, Freeman and colleagues showed that engagement of FcγR increased the lateral membrane mobility of CD45 due to loss of cytoskeletal constraint, yet CD45 was found to be excluded from the nascent phagocytic cup *via* an integrin-dependent barrier. The depletion of CD45 from the phagocytic-target interface was shown to facilitate phagocytosis. Thus, the formation of an integrin-dependent diffusional barrier acted to increase the efficiency of phagocytosis at low levels of opsonization (Freeman et al., 2016). We reported that cross-linking of macrophage CD44 acted to rapidly augment phagocytosis of apoptotic neutrophils (Hart et al., 1997). Although the mechanism remains to be fully defined, cytoskeletal reorganization observed following CD44 cross-linking, including Rac activation, altered podosome formation, and migratory capacity (Hart et al., 2012), would be consistent with changes in the extent of CD44-dependent restriction of lateral membrane receptor movement (Freeman et al., 2018).

Ligation of other macrophage receptors may also act to increase phagocytosis of ACs. Zizzo and Cohen demonstrated that antibody-induced cross-linking of CD14 promoted phosphorylation of Mer receptor tyrosine kinase (Mer) and potentiated phagocytosis of ACs (Zizzo and Cohen, 2018). The presence of exogenous beta galactoside binding lectin, galectin-3, acts to promote phagocytosis of ACs (Erriah et al., 2019), possibly *via* cross-linking of the integrin αVβ3 (Jiang et al., 2012). Galectin-3 has also been reported to bind to Mer (Caberoy et al., 2012).

## Recognition of PtdSer *via* Tyro3, Axl, and Mer Receptor Tyrosine Kinases

Mer and the related receptor tyrosine kinases Tyro3 and Axl enable phagocytes to recognize PtdSer exposed on the surface of AC *via* binding to the PtdSer opsonins Protein S and Gas6 (Lemke, 2013). Although signaling downstream of Mer promotes cytoskeletal rearrangements that are necessary for internalization (Tibrewal et al., 2008), Mer signaling also has an important role in the resolution of inflammation by driving production of specialized resolving mediators, including LxA4 and RvD1 (Cai et al., 2016; Cai et al., 2018). Mer-deficient mice exhibit impaired phagocytosis of ACs contributing to development of allergic inflammation (Felton et al., 2018), atherosclerosis (Thorp et al., 2008), or autoimmune diseases (Rothlin et al., 2015). Proteases that are likely present at sites of inflammation can reduce the activity of Mer. Specific cleavage of Mer from the phagocyte surface was demonstrated to be mediated by ADAM17 (Thorp et al., 2011) reduced AC phagocytosis and may represent a key mechanism controlling AC clearance capacity during progression of inflammatory responses (Lee et al., 2012; Cai et al., 2017). In addition, the presence of soluble Mer may compete for phagocyte binding to protein S or Gas6 opsonized AC, decreasing Mer-dependent phagocytosis as a consequence (Sather et al., 2007). Inhibition of proteolytic cleavage of Mer was shown to ameliorate LPS-induced lung injury (Lee et al., 2012), and in animals expressing a cleavage-resistant form of Mer, inflammation-mediated tissue damage was reduced with improved resolution of inflammation (DeBerge et al., 2017a).

## Regulation of AC Phagocytosis by Glucocorticoids

The capacity for phagocytosis of AC is altered during differentiation and activation of macrophages. In particular, acquisition of a macrophage phenotype associated with tissue repair correlates with increased phagocytosis of AC. For example, glucocorticoids (including methylprednisolone, hydrocortisone, and dexamethasone) are potent drivers for the engulfment of AC (Liu et al., 1999; Giles et al., 2001), inducing marked changes in the receptor expression profile of macrophages. Over 100 genes have been shown to be modulated by glucocorticoids including receptors involved in phagocytosis of ACs such as CD163, FPR1, and Mer (Ehrchen et al., 2007). Glucocorticoid-mediated alterations in the macrophage phenotype also include inhibition of release of pro-inflammatory cytokines together with anti-inflammatory cytokine production (e.g. TGFβ, IL-10 and IL-1ra), thereby promoting tissue repair and regeneration. In addition, downregulation or reduced phosphorylation of key integrin signaling molecules such as paxillin, pyk2, and p130Cas (Giles et al., 2001) may limit focal adhesion formation, allowing integrins to participate in phagocytosis of ACs. In addition, glucocorticoid-induced upregulation of expression of the integrin ligand MFG-E8 may further contribute to augmentation of AC phagocytosis. Deficiency of MFG-E8 or knockdown with RNAi reduced the extent of AC phagocytosis following treatment with glucocorticoids (Lauber et al., 2013). Augmented AC phagocytosis following glucocorticoid treatment was shown to be primarily dependent on Mer and the Mer ligand, protein S (McColl et al., 2009; Zizzo et al., 2012). Increased expression of Mer following glucocorticoid treatment confers the capacity for tethering and subsequent phagocytosis of AC by macrophages (Dransfield et al., 2015). In keeping with these findings, increased phagocytosis of apoptotic cells following GC treatment was not observed in macrophages from Mer knockout mice (Zagórska et al., 2014). The increased protein S-dependent phagocytosis of AC by macrophages following glucocorticoid treatment was shown to be reversed by interferon-γ (Heasman et al., 2004). This observation may be explained by the strong induction of Axl in the presence of pro-inflammatory cytokines such as interferon-γ, leading to engagement of Axl-dependent phagocytosis (Zagórska et al., 2014), which, in contrast to Mer, is not mediated by protein S.

## Regulation of AC Phagocytosis by Other Nuclear Receptors

Activation of the nuclear receptors, LXR, and the peroxisome proliferator-activated receptors (PPAR) γ and δ also upregulates AC phagocytic capacity (A-Gonzalez and Hidalgo, 2014), providing a mechanism for sensing the uptake of apoptotic material and enhancement of the phagocytic capacity accordingly. Specific deletion or downregulation of LXRα/β (A-Gonzalez et al., 2009) and PPARδ and retinoid X receptor markedly reduces phagocytosis efficiency (Mukundan et al., 2009). As described for glucocorticoids above, LXR agonists were found to induce expression of Mer leading to augmentation of phagocytosis (A-Gonzalez et al., 2009). Regulation of macrophage inflammatory pathways by the LXR agonist GW3965 significantly attenuated the clinical and histological severity in a model of collagen-induced arthritis in mice (Park et al., 2010). Inflammatory mediator production within the joint and serum pro-inflammatory cytokine levels were inhibited, raising the possibility that targeting LXR may represent a therapeutic target to reduce the severity of joint destruction in rheumatoid arthritis.

Inhibition of PPAR-γ, using the antagonist GW9662, inhibited LPS-induced IL-10 production and decreased AC phagocytosis, in part *via* downregulation of the key phagocytic receptors CD36, transglutaminase-2, and Axl (Majai et al., 2007; Zizzo and Cohen, 2015). Antagonism of PPAR-γ was also found to promote macrophage differentiation and upregulation of Mer-dependent AC phagocytosis. In contrast, upregulation of Mer expression was blocked by the PPAR-γ agonist rosiglitazone, suggesting that PPAR-γ negatively controls the expansion of anti-inflammatory macrophages that exhibit efficient AC phagocytosis (Zizzo and Cohen, 2015).

## Phagocyte PtdSer and Engulfment

It is well established that PtdSer exposure on the AC surface represents a near universal cue that signals phagocyte recognition (Segawa and Nagata, 2015). However, transient exposure of PtdSer on the phagocyte membrane facilitates phagocytosis *via* alteration of the local membrane environment. Callahan and co-workers showed that Annexin V and the lipid binding dye merocyanine (that binds strongly to AC) also bound specifically to non-apoptotic macrophages (Callahan et al., 2000), a finding also confirmed in *C. elegans* (Mapes et al., 2012). Redistribution of PtdSer to outer leaflet is promoted by the ATP binding cassette transporter ABCA1 (Marguet et al., 1999), and ABCA1-deficient cells are less efficient phagocytes (Hamon et al., 2000). Uptake of ACs also acts to provide a positive amplification loop, inducing expression of ABCA1 *via* LXR-dependent and -independent pathways (Fond et al., 2015; Kiss et al., 2006) leading to further increase of cholesterol efflux. Lyso-PtdSer induced G2A receptor signaling in macrophages to enhance production of prostaglandin E2 (PGE2), activation of EP2 receptors, and adenylate cyclase resulting in cAMP elevation and Rac activation (Frasch et al., 2011). This effect was dynamic, with low levels of cAMP augmenting phagocytosis of AC, whereas high levels were inhibitory, in keeping with previously published work (Rossi et al., 1998).

## Extracellular Vesicles and Regulation of AC Phagocytosis

A role for extracellular vesicles (EV) in phagocyte exposure of PtdSer was suggested by studies in *C. elegans* where Ced-7, TTR-52 (PtdSer binding protein), and Ced-1 were required (Mapes et al., 2012). Vesicles derived from AC (apoEV) may have multiple immunomodulatory effects (Lynch et al., 2017) that may be dependent on the specific membrane composition and vesicle contents, or cargo (Caruso and Poon, 2018). EV originating from different cellular sources may have distinct surface profiles that exert differential effects on macrophage function. It has been shown that apoEVs may facilitate AC phagocytosis by presentation of molecules such as ICAM-3 that specifically direct the recruitment of phagocytes (Torr et al., 2012). Exposure of PtdSer on the EV surface allows engagement of receptors involved in AC uptake (Mohning et al., 2018), including the Axl-Gas6 pathway for platelet-derived EV (Happonen et al., 2016). Phagocytosis may be further augmented in the presence of apoEV, suggesting that EV exert direct regulatory effects on macrophage functional activity (Distler et al., 2005). It is now clear that EV exert control of macrophage function that may critically determine the course of an inflammatory response. In addition, the inflammatory microenvironment may critically determine macrophage responses to EV. Type I interferons were reported to promote phagocytosis of apoEV, leading to acquisition of a pro-inflammatory macrophage phenotype (Niessen et al., 2015). Interaction of phagocytes with EV derived from non-activated neutrophils was shown to inhibit pro-inflammatory cytokine production (Eken et al., 2013), contrasting the potentially pathogenic effects of EV derived from activated neutrophils (Genschmer et al., 2019). Specific EV contents could further regulate reprogramming of macrophage behavior as found for platelet-derived EV delivery of miR126 (Laffont et al., 2016). Finally, it has been shown that apoEV may acquire distinct lipid profiles by binding to specialized pro-resolving mediators, including RvD1 (Dalli and Serhan, 2012). This distinct lipid profile was shown to increase macrophage phagocytosis in a GPCR-dependent manner, *via* a mechanism that involves production of RvD2, MaR1, PGE2, and PGF2 by macrophages (Dalli and Serhan, 2012).

## Other Factors

In general terms, the capacity for macrophage phagocytosis of different particles is dependent on target size and the presence of opsonizing ligands, including antibodies or serum factors (Cannon and Swanson, 1992). Early studies suggested that for macrophage phagocytosis of AC, prior uptake inhibited further phagocytic capacity (Erwig et al., 1999) possibly as a result of reduced membrane availability following internalization of a large AC target. The source of lipids that macrophages utilized for uptake of multiple targets (Gagnon et al., 2002) was proposed to be the endoplasmic reticulum (Duclos et al., 2003), but this was not confirmed in subsequent detailed studies (Touret et al., 2005). Continued uptake of AC by phagocytes may depend on metabolic status. For example, reduced mitochondrial membrane potential was found to increase phagocytic capacity, with a pivotal role for the Ucp2 protein (Park et al., 2011). In *Drosophila melanogaster*, fragments of ACs were shown to enhance subsequent AC phagocytosis. The mechanism appeared to involve activation of Tailless upregulation of Draper and PS3 integrin to enhance phagocytic activity (Nonaka et al., 2017). It is now clear that phagocytosis of AC also causes intracellular changes that impact upon macrophage function. Macrophages taking up AC were found to have reduced mitochondrial length, with increased expression of molecules involved in regulating mitochondrial fission, such as Drp1 (Wang et al., 2017). In drp1−/− macrophages, early ingestion of ACs was found to be unaffected, but later AC phagocytosis was reduced compared to control. Since Drp1 inhibition also reduced AC phagocytosis at later timepoints, mitochondrial fission was suggested to enable multiple AC uptake. In the absence of mitochondrial fission, AC induced Ca^2+^ responses are impaired, reducing subsequent phagosome formation (Wang et al., 2017).

## Negative Regulators

Phagocytosis of AC is critically dependent on the balance of activity of the Rac/Rho/cdc42 family of small GTPases. Macrophages actively extend actin-rich processes to “explore” their surroundings (Flannagan et al., 2010). There is some evidence that Rho and Rac may be inversely co-regulated in phagocytes. Whereas RhoA negatively regulates phagocytosis of AC, Rac-1 activation enables efficient uptake, leading to formation of phagocytic “portals” (Nakaya et al., 2008). These AC portals, often associated with lamellipodia, allow multiple targets to be internalized at the same site. However, constitutive Rac1 activation delays phagocytic cup closure and inhibits phagocytosis. Downregulation of CD47 provides a mechanism to promote phagocytosis of ACs (Lawrence et al., 2009). Expression of CD47 on viable cells acts to inhibit phagocytosis by binding to the counter-receptor SIRPα expressed on the phagocyte membrane (Tsai and Discher, 2008; Lv et al., 2015). SIRPα-dependent activation of the tyrosine phosphatase SHP1/2 signaling results in inhibition of Rac1 activation (Oldenborg et al., 2001). This mechanism for prevention of phagocytosis of viable cells by negative regulators such as CD47 should represent an important control pathway in tissue homeostasis. However, mice lacking CD47 exhibit enhanced susceptibility to infection and reduced recruitment of granulocytes (Lindberg et al., 1996); no other major phenotype was noted. It appears that the CD47-SIRPα regulatory pathway is indispensable for controlling the extent of self-phagocytosis in a tissue environment where pro-inflammatory conditions drive macrophage phagocytic responses (Bian et al., 2016). Therapeutic targeting of the CD47 pathway may be particularly important in diseases where phagocytosis of AC has been compromised. For example, antibody-mediated blockade of CD47 was found to restore defective AC clearance in atherosclerotic lesions, resulting in reduced atherosclerotic burden (Kojima et al., 2016). Since elevated expression of CD47 on tumor cells may promote tumor growth by providing an immune escape mechanism, blocking the CD47 pathway may provide a strategy for driving phagocyte destruction of tumor cells (Matlung et al., 2017).

## Therapeutic Implications

In health, non-internalized AC are present at very low levels within tissues (Kerr et al., 1972) suggesting that AC clearance capacity is matched to the overall tissue load of AC. Following injury or infection, imbalances between the rates of apoptosis and phagocytic clearance would result in accumulation of AC within tissues. Although the presence of AC may be a normal feature of physiological responses to tissue injury, AC may eventually undergo secondary necrosis and contribute to perpetuation of inflammatory responses associated with disease. Although therapeutic strategies targeting the apoptotic process may be sufficient to drive resolution of inflammation (Cartwright et al., 2019), modulation of phagocytosis of AC may provide additional options for promotion of repair and restoration of tissue homeostasis following injury or infection. One potential mechanism for regulating phagocytosis of cells would be downregulation of “don’t eat me” signals on AC targets (Barclay and Van den Berg, 2014). However, given the potential for off-target effects, we will instead consider the potential for exploiting the regulatory mechanisms controlling AC phagocytosis.

First, the differentiation/activation status of macrophages present at inflamed sites could be altered to induce expression of pro-phagocytic receptors. For example, treatment with glucocorticoids (McColl et al., 2007) or LXR agonists (A-Gonzalez et.al, 2009) would upregulate expression of receptors that are involved in AC clearance such as Mer (Röszer, 2017) and hence promote phagocytic clearance of AC. An alternative approach might be to exploit miRNA that specifically regulates phagocytic receptors. Kurowska-Stolarska et al. (2017) showed that miR-34 acted to reduce expression of Axl, and that targeting miR-34 reduced pro-inflammatory cytokine production and dendritic cell activation in mice (Kurowska-Stolarska et al., 2017). The availability of ligands that allow “bridging” of phagocytes to AC targets may be critical. For example, protein S likely represents a key mechanism for reprogramming macrophage maturation, with increased pro-inflammatory mediators (TNF) and reduced anti-inflammatory mediators RvD1 and IL-10 in the protein S-deficient mice (Lumbroso et al., 2018). In terms of therapy, overexpression of the Mer ligands protein S and Gas-6 has been shown to reduce inflammation (ankle swelling, pro-inflammatory cytokine levels) in a collagen-induced model of arthritis in mice (van den Brand et al., 2013). Exogenous administration of AC bridging ligands might have therapeutic benefit in a range of inflammatory diseases.

Second, the action of proteases that regulate the expression of phagocytic receptors could be blocked. Inhibitors of ADAM-17 or MMP9 would be predicted to reduce proteolytic downregulation of Mer, LRP1, SRB-1, or CD36 shedding that is associated with reduced capacity for phagocytosis of AC and development of disease, for example in atherosclerotic lesions (DeBerge et al., 2017b). In diabetic mice, high levels of glucose downregulated MiR-126 leading to increased expression of ADAM9 and suppression of Mer-dependent phagocytosis of AC (Suresh Babu et al., 2016). Overexpression of miR-126 conferred rescue of phagocytic defects in response to environmental conditions that suppress AC phagocytosis, such as high glucose (Suresh Babu et al., 2016).

Third, stimulation of GPCRs mediating the effects of pro-resolving mediators may provide a rapid mechanism for the regulation of phagocytosis of AC.

Finally, microvesicles with defined membrane lipids or protein repertoires may represent a useful mechanism for the modulation of macrophage function (Gregory and Pound, 2011). Microvesicles that express accessible PtdSer might be opsonized with PtdSer bridging ligands or specialized pro-resolving mediators, providing a combination of signals that mimics the AC surface. Such tailored microvesicles could be administered directly to sites of injury or inflammation and act to promote acquisition of a macrophage phenotype that is pro-resolution.

In summary, AC clearance is a key process in the control of tissue repair and regeneration. Strategies to overcome defective clearance of apoptotic material could provide new approaches to treating established inflammatory or autoimmune diseases.

## Author Contributions

ID conceived and wrote and edited the manuscript and prepared the tables; SA and NDB wrote the manuscript; DAD and AGR edited the manuscript.

## Funding

The authors would like to acknowledge funding from the Medical Research Council UK (MR/KO13386/1: AGR), Engineering and Physical Sciences Research Council and MRC Centre for Doctoral Training in Optical Imaging (OPTIMA) (EP/L016559; NDB), and the Medical Research Foundation National PhD Programme in Antimicrobial Resistance (SA).

## Conflict of Interest Statement

The authors declare that the research was conducted in the absence of any commercial or financial relationships that could be construed as a potential conflict of interest.
